# Current Awareness on Comparative and Functional Genomics

**DOI:** 10.1002/cfg.350

**Published:** 2004-02


**Comparative and Functional Genomics**
*Comp Funct Genom* 2004; **5**: 108–114
Published online in Wiley InterScience (www.interscience.wiley.com). **DOI:** 10.1002/cfg.350

### Current awareness on comparative and functional genomics

### 1 Reviews & symposia

2003. Special issue on bioinformatics software. *Nucleic Acids Res* **31:** (13)
2003. Special issue: Functional genomics and proteomics in control of breathing. *Respir Physiol Neurobiol* **135:** (2-3)
2003. Special issue: Genomewide surveys of developmentally relevant genes in *Ciona intestinalis*. *Dev Genes Evol* **213:** (5-6)
Aebersold R. 2003. Quantitative proteome analysis: Methods and applications. *J Infect Dis* **187:** (Suppl 2) S315.
Angeletti C. 2003. Application of proteomic technologies to cytologic specimens - A review. *Acta Cytol* **47:** (4) 535.
Arthur JM. 2003. Proteomics. *Curr Opin Nephrol Hypertens* **12:** (4) 423.
Ashcroft AE. 2003. Protein and peptide identification: The role of mass spectrometry in proteomics. *Nat Prod Rep* **20:** (2) 202.
Bader GD, Heilbut A, Andrews B, Tyers M, Hughes T, Boone C. 2003. Functional genomics and proteomics: Charting a multidimensional map of the yeast cell. *Trends Cell Biol* **13:** (7) 344.
Bradbury A, Velappan N, Verzillo V, Ovecka M, Chasteen L, Sblattero D, Marzari O, Lou JL, Siegel R, Pavlik P. 2003. Antibodies in proteomics II: Screening, high-throughput characterization and downstream applications (Review). *Trends Biotechnol* **21:** (7) 312.
Burger G, Lang BF. 2003. Parallels in genome evolution in mitochondria and bacterial symbionts. *IUBMB Life* **55:** (4-5) 205.
Chandler JW, Werr W. 2003. When negative is positive in functional genomics (Review). *Trends Plant Sci* **8:** (6) 279.
Dow JAT, Davies SA. 2003. Integrative physiology and functional genomics of epithelial function in a genetic model organism. *Physiol Rev* **83:** (3) 687.
Ellis JT, Morrison DA, Reichel MP. 2003. Genomics and its impact on parasitology and the potential for development of new parasite control methods. *DNA Cell Biol* **22:** (6) 395.
Flugge UI, Hausler RE, Ludewig F, Fischer K. 2003. Functional genomics of phosphate antiport systems of plastids (Review). *Physiol Plant* **118:** (4) 475.
Hayden PS, El-Meanawy A, Schelling JR, Sedor JR*. 2003. DNA expression analysis: Serial analysis of gene expression, microarrays and kidney disease. *Curr Opin Nephrol Hypertens* **12:** (4) 407.
Irish VF. 2003. The evolution of floral homeotic gene function (Review). *Bioessays* **25:** (7) 637.
Janin J, Seraphin B. 2003. Genome-wide studies of protein-protein interaction. *Curr Opin Struct Biol* **13:** (3) 383.
Jedrzejas MJ, Huang WJM. 2003. *Bacillus* species proteins involved in spore formation and degradation: From identification in the genome, to sequence analysis, and determination of function and structure. *Crit Rev Biochem Molec Biol* **38:** (3) 173.
Joos L, Eryuksel E, Brutsche MH*. 2003. Functional genomics and gene microarrays: The use in research and clinical medicine (Review). *Swiss Med Wkly* **133:** (3-4) 31.
Karlin S, Mrazek J, Gentles AJ. 2003. Genome comparisons and analysis. *Curr Opin Struct Biol* **13:** (3) 344.
Kinoshita K, Nakamura H. 2003. Protein informatics towards function identification. *Curr Opin Struct Biol* **13:** (3) 396.
Lake S, Krook A, Zierath JR*. 2003. Analysis of insulin signaling pathways through comparative genomics. Mapping mechanisms for insulin resistance in type 2 (non-insulin-dependent) diabetes mellitus (Review). *Exp Clin Endocrinol Diabetes* **111:** (4) 191.
Lee D, Grant A, Buchan D, Orengo C. 2003. A structural perspective on genome evolution. *Curr Opin Struct Biol* **13:** (3) 359.
Li Y, Lambert MH, Xu HE*. 2003. Activation of nuclear receptors: A perspective from structural genomics (Review). *Structure* **11:** (7) 741.
Lin BC, Vahey MT, Thach D, Stenger DA, Pancrazio JJ. 2003. Biological threat detection *via* host gene expression profiling (Review). *Clin Chem* **49:** (7) 1045.
Maguire PB, Fitzgerald DJ. 2003. Platelet proteomics. *J Thromb Haemost* **1:** (7) 1593.
Man O, Atarot T, Sadot A, Olender T, Lancet D. 2003. From subgenome analysis to protein structure. *Curr Opin Struct Biol* **13:** (3) 353.
Matte A, Sivaraman J, Ekiel I, Gehring K, Jia Z, Cygler M. 2003. Contribution of structural genomics to understanding the biology of *Escherichia coli* (Review). *J Bacteriol* **185:** (14) 3994.
McDonald TG, Van Eyk JE*. 2003. Mitochondrial proteomics: Undercover in the lipid bilayer. *Basic Res Cardiol* **98:** (4) 219.
Morrison DA, Ellis JT*. 2003. The design and analysis of microarray experiments: Applications in parasitology. *DNA Cell Biol* **22:** (6) 357.
Orchard S, Hermjakob H*, Apweiler R. 2003. The proteomics standards initiative. *Proteomics* **3:** (7) 1374.
Petricoin EF, Liotta LA. 2003. Clinical applications of proteomics. *J Nutr* **133:** (7) 2476S.
Puente XS, Sanchez LM, Overall CM, Lopez-Otin C*. 2003. Human and mouse proteases: A comparative genomic approach. *Nat Rev Genet* **4:** (7) 544.
Romijn EP, Krijgsveld J, Heck AJR*. 2003. Recent liquid chromatographic-(tandem) mass spectrometric applications in proteomics. *J Chromatogr A* **1000:** (1-2) 589.
Salwinski L, Eisenberg D. 2003. Computational methods of analysis of protein-protein interactions. *Curr Opin Struct Biol* **13:** (3) 377.
Schloss PD, Handelsman J*. 2003. Biotechnological prospects from metagenomics. *Curr Opin Biotechnol* **14:** (3) 303.
Shaughnessy JD, Barlogie B. 2003. Interpreting the molecular biology and clinical behavior of multiple myeloma in the context of global gene expression profiling. *Immunol Rev* **194:** (1) 140.
Stolovitzky G. 2003. Gene selection in microarray data: The elephant, the blind men and our algorithms. *Curr Opin Struct Biol* **13:** (3) 370.
Watson JD, Todd AE, Bray J, Laskowski RA, Edwards A, Joachimiak A, Orengo CA, Thornton JM*. 2003. Target selection and determination of function in structural genomics. *IUBMB Life* **55:** (4-5) 249.
Westermeier R, Loyland S, Asbury R. 2002. Proteomics technology. *J Clin Ligand Assay* **25:** (3) 242.

### 3 Large-scale sequencing and mapping

Garnier T, Eiglmeier K, Camus JC, Medina N, Mansoor H, Pryor M, Duthoy S, Grondin S, Lacroix C, Monsempe C *et al*. 2003. The complete genome sequence of *Mycobacterium bovis*. *Proc Natl Acad Sci U S A* **100:** (13) 7877.
Glockner FO, Kube M, Bauer M, Teeling H, Lombardot T, Ludwig W, Gade D, Beck A, Borzym K, Heitmann K *et al*. 2003. Complete genome sequence of the marine planctomycete *Pirellula* sp strain 1. *Proc Natl Acad Sci U S A* **100:** (14) 8298.
Maglich JM, Caravella JA, Lambert MH, Willson TM, Moore JT*, Ramamurthy L. 2003. The first completed genome sequence from a teleost fish (*Fugu rubripes*) adds significant diversity to the nuclear receptor superfamily. *Nucleic Acids Res* **31:** (14) 4051.
Suerbaum S, Josenhans C, Sterzenbach T, Drescher B, Brandt P, Bell M, Droge M, Fartmann B, Fischer HP, Ge ZM *et al*. 2003. The complete genome sequence of the carcinogenic bacterium *Helicobacter hepaticus*. *Proc Natl Acad Sci U S A* **100:** (13) 7901.

In order to keep subscribers up-to-date with the latest developments in their field, this current awareness service is provided by John Wiley & Sons and contains newly-published material on comparative and functional genomics. Each bibliography is divided into 16 sections. 1 Reviews & symposia; 2 General; 3 Large-scale sequencing and mapping; 4 Evolutionary genomics; 5 Comparative genomics; 6 Pathways, gene families and regulons; 7 Pharmacogenomics; 8 EST, cDNA and other clone resources; 9 Functional genomics; 10 Transcriptomics; 11 Proteomics; 12 Protein structural genomics; 13 Metabolomics; 14 Genomic approaches to development; 15 Technological advances; 16 Bioinformatics. Within each section, articles are listed in alphabetical order with respect to author. If, in the preceding period, no publications are located relevant to any one of these headings, that section will be omitted.

Copyright © 2004 John Wiley & Sons, Ltd.

Volik S, Zhao SY, Chin K, Brebner JH, Herndon DR, Tao QZ, Kowbel D, Huang GQ, Lapuk A, Kuo WL *et al*. 2003. End-sequence profiling: Sequence-based analysis of aberrant genomes. *Proc Natl Acad Sci U S A* **100:** (13) 7696.

### 4 Evolutionary genomics

Kunin V, Ouzounis CA*. 2003. The balance of driving forces during genome evolution in prokaryotes. *Genome Res* **13:** (7) 1589.
Michalak P, Noor MAF. 2003. Genome-wide patterns of expression in *Drosophila* pure species and hybrid males. *Mol Biol Evol* **20:** (7) 1070.
Mineta K, Nakazawa M, Cebria F, Ikeo K, Agata K, Gojobori T. 2003. Origin and evolutionary process of the CNS elucidated by comparative genomics analysis of planarian ESTs. *Proc Natl Acad Sci U S A* **100:** (13) 7666.
Pevzner P, Tesler G*. 2003. Human and mouse genomic sequences reveal extensive breakpoint reuse in mammalian evolution. *Proc Natl Acad Sci U S A* **100:** (13) 7672.
Riehle MM, Bennett AF, Lenski RE, Long AD. 2003. Evolutionary changes in heat-inducible gene expression in lines of *Escherichia coli* adapted to high temperature. *Physiol Genomics* **14:** (1) 47.
Wolfgang MC, Kulasekara BR, Liang XY, Boyd D, Wu K, Yang Q, Miyada CG, Lory S*. 2003. Conservation of genome content and virulence determinants among clinical and environmental isolates of *Pseudomonas aeruginosa*. *Proc Natl Acad Sci U S A* **100:** (14) 8484.

### 5 Comparative genomics

Jaillon O, Dossat C, Eckenberg R, Eiglmeier K, Segurens A, Aury JM, Roth CW, Scarpelli C, Brey PT, Weissenbach J *et al*. 2003. Assessing the *Drosophila melanogaster* and *Anopheles gambiae* genome annotations using genome-wide sequence comparisons. *Genome Res* **13:** (7) 1595.
Karaman MW, Houck ML, Chemnick LG, Nagpal S, Chawannakul D, Sudano D, Pike BL, Ho VV, Ryder OA, Hacia JG*. 2003. Comparative analysis of gene-expression patterns in human and African great ape cultured fibroblasts. *Genome Res* **13:** (7) 1619.
Li G, Gao M, Yang B, Quiros CF. 2003. Gene for gene alignment between the *Brassica* and *Arabidopsis* genomes by direct transcriptome mapping. *Theor Appl Genet* **107:** (1) 168.
McCutcheon JP, Eddy SR*. 2003. Computational identification of non-coding RNAs in *Saccharomyces cerevisiae* by comparative genomics. *Nucleic Acids Res* **31:** (14) 4119.
Nishio Y, Nakamura Y, Kawarabayasi Y, Usuda Y, Kimura E, Sugimoto S, Matsui K, Yamagishi A, Kikuchi H, Ikeo K *et al*. 2003. Comparative complete genome sequence analysis of the amino acid replacements responsible for the thermostability of *Corynebacterium efficiens*. *Genome Res* **13:** (7) 1572.
Nishiyama T, Fujita T, Shinl T, Seki M, Nishide H, Uchiyama I, Kamiya A, Carninci P, Hayashizaki Y, Shinozaki K *et al*. 2003. Comparative genomics of *Physcomitrella patens* gametophytic transcriptome and *Arabidopsis thaliana*: Implication for land plant evolution. *Proc Natl Acad Sci U S A* **100:** (13) 8007.

### 6 Pathways, gene families and regulons

Barrangou R, Altermann E, Hutkins R, Cano R, Klaenhammer TR. 2003. Functional and comparative genomic analyses of an operon involved in fructooligosaccharide utilization by *Lactobacillus acidophilus*. *Proc Natl Acad Sci U S A* **100:** (15) 8957.
Deschamps S, Meyer J, Chatterjee G, Wang H, Lengyel P, Roe BA*. 2003. The mouse *Ifi200* gene cluster: Genomic sequence, analysis, and comparison with the human *HIN-200* gene cluster. *Genomics* **82:** (1) 34.
Papp B, Pal C, Hurst LD*. 2003. Dosage sensitivity and the evolution of gene families in yeast. *Nature* **424:** (6945) 194.
Ravasi T, Huber T, Zavolan M, Forrest A, Gaasterland T, Grimmond S, Hume DA. 2003. Systematic characterization of the zinc-finger-containing proteins in the mouse transcriptome. *Genome Res* **13:** (6B) 1430.
Tajul-Arifin K, Teasdale R, Ravasi T, Hume DA, Mattick JS. 2003. Identification and analysis of chromodomain-containing proteins encoded in the mouse transcriptome. *Genome Res* **13:** (6B) 1416.

### 7 Pharmacogenomics

Abe M, Yamashita S, Kuramoto T, Hirayama Y, Tsukamoto T, Ohta T, Tatematsu M, Ohki M, Takato T, Sugimura T *et al*. 2003. Global expression analysis of *N*-methyl-*N*-nitro-*N*-nitrosoguanidine-induced rat stomach carcinomas using oligonucleotide microarrays. *Carcinogenesis* **24:** (5) 861.
Ahn SK, Choe TB, Kwon TJ*. 2003. The gene expression profile of human umbilical vein endothelial cells stimulated with lipopolysaccharide using cDNA microarray analysis. *Int J Mol Med* **12:** (2) 231.
Augenlicht LH, Velcich A, Klampfer L, Huang J, Corner G, Aranes M, Laboisse C, Rigas B, Lipkin M, Yang K *et al*. 2003. Application of gene expression profiling to colon cell maturation, transformation and chemoprevention. *J Nutr* **133:** (7) 2410S.
Boraldi F, Bini L, Liberatori S, Armini A, Pallini V, Tiozzo R, Pasquali-Ronchetti I, Quaglino D*. 2003. Proteome analysis of dermal fibroblasts cultured *in vitro* from human healthy subjects of different ages. *Proteomics* **3:** (6) 917.
Cappola TP, Cope L, Cernetich A, Barouch LA, Minhas K, Irizarry RA, Parmigiani G, Durrani S, Lavoie T, Hoffman EP *et al*. 2003. Deficiency of different nitric oxide synthase isoforms activates divergent transcriptional programs in cardiac hypertrophy. *Physiol Genomics* **14:** (1) 25.
Carinci F, Francioso F, Piattelli A, Rubini C, Fioroni M, Evangelisti R*, Arcelli D, Tosi L, Pezzetti F, Carinci P *et al*. 2003. Genetic expression profiling of six odontogenic tumors. *J Dent Res* **82:** (7) 551.
Catalano RD, Yanaihara A, Evans AL, Rocha D, Prentice A, Saidi S, Print CG, Charnock-Jones DS, Sharkey AM, Smith SK. 2003. The effect of RU486 on the gene expression profile in an endometrial explant model. *Mol Hum Reprod* **9:** (8) 465.
Cecconi D, Scarpa A, Donadelli M, Palmieri M, Hamdan M, Astner H, Righetti PG*. 2003. Proteomic profiling of pancreatic ductal carcinoma cell lines treated with trichostatin-A. *Electrophoresis* **24:** (11) 1871.
Chang JC, Wooten EC, Tsimelzon A, Hilsenbeck SG, Gutierrez MC, Elledge R, Mohsin S, Osborne CK, Chamness GC, Allred DC *et al*. 2003. Gene expression profiling for the prediction of therapeutic response to docetaxel in patients with breast cancer. *Lancet* **362:** (9381) 362.
Chaudhry MA, Chodosh LA, McKenna WG, Muschel RJ. 2003. Gene expression profile of human cells irradiated in $G_1$ and $G_2$ phases of cell cycle. *Cancer Lett* **195:** (2) 221.
Chaussabel D, Semnani RT, McDowell MA, Sacks D, Sher A, Nutman TB*. 2003. Unique gene expression profiles of human macrophages and dendritic cells to phylogenetically distinct parasites. *Blood* **102:** (2) 672.
Da Silva L, Cote D, Roy C, Martinez M, Duniho S, Pitt MLM, Downey T, Dertzbaugh M. 2003. Pulmonary gene expression profiling of inhaled ricin. *Toxicon* **41:** (7) 813.
De Jonge RR, Vreijling JP, Meintjes A, Kwa MSG, Van Kampen AHC, Van Schaik IN, Baas F*. 2003. Transcriptional profile of the human peripheral nervous system by serial analysis of gene expression. *Genomics* **82:** (2) 97.
Delcayre A, Peake JS, White DJ, Yuan SN, McDonald MK, Liang A, Tan PL, Watson JD. 2003. A genome-based functional screening approach to vaccine development that combines *in vitro* assays and DNA immunization. *Vaccine* **21:** (23) 3259.
Diamandis EP. 2003. Proteomic patterns in biological fluids: Do they represent the future of cancer diagnostics? *Clin Chem* **49:** (8) 1272.
Duran MC, Mas S, Martin-Ventura JL, Meilhac O, Michel JB, Gallego-Delgado J, Lazaro A, Tunon J, Egido J, Vivanco F. 2003. Proteomic analysis of human vessels: Application to atherosclerotic plaques. *Proteomics* **3:** (6) 973.
Fratelli M, Demol H, Puype M, Casagrande S, Villa P, Eberini I, Vandekerckhove J, Gianazza E, Ghezzi P. 2003. Identification of proteins undergoing glutathionylation in oxidatively stressed hepatocytes and hepatoma cells. *Proteomics* **3:** (7) 1154.
Ghafouri B, Tagesson C, Lindahl M. 2003. Mapping of proteins in human saliva using two-dimensional gel electrophoresis and peptide mass fingerprinting. *Proteomics* **3:** (6) 1003.
Gineste C, Ho L, Pompl P, Bianchi M, Pasinetti GM. 2003. High-throughput proteomics and protein biomarker discovery in an experimental model of inflammatory hyperalgesia: Effects of nimesulide. *Drugs* **63:** (Suppl 1) 23.

Copyright © 2004 John Wiley & Sons, Ltd.

Grus FH, Joachim SC, Pfeiffer N. 2003. Analysis of complex autoantibody repertoires by surface-enhanced laser desorption/ionization-time of flight mass spectrometry. *Proteomics* **3:** (6) 957.

Hansel DE, Rahman A, Hidalgo M, Thuluvath PJ, Lillemoe KD, Shulick R, Ku JL, Park JG, Miyazaki K, Ashfaq R *et al*. 2003. Identification of novel cellular targets in biliary tract cancers using global gene expression technology. *Am J Pathol* **163:** (1) 217.

Hashimoto SI, Nagai S, Sese J, Suzuki T, Obata A, Sato T, Toyoda N, Dong HY, Kurachi M, Nagahata T, Shizuno K, Morishita S, Matsushima K. 2003. Gene expression profile in human leukocytes. *Blood* **101:** (9) 3509.

Henshall SM, Afar DEH, Hiller J, Horvath LG, Quinn DI, Rasiah KK, Gish K, Willhite D, Kench JG, Gardiner-Garden M *et al*. 2003. Survival analysis of genome-wide gene expression profiles of prostate cancer identifies new prognostic targets of disease relapse. *Cancer Res* **63:** (14) 4196.

Hernandez A, Karrow N, Mallard BA. 2003. Evaluation of immune responses of cattle as a means to identify high or low responders and use of a human microarray to differentiate gene expression. *Genet Sel Evol* **35:** (Suppl 1) S67.

Hsieh HL, Schafer BW, Sasaki N, Heizmann CW. 2003. Expression analysis of S100 proteins and RAGE in human tumors using tissue microarrays. *Biochem Biophys Res Commun* **307:** (2) 375.

Ichiba T, Teshima T, Kuick R, Misek DE, Liu C, Takada Y, Maeda Y, Reddy P, Williams DL, Hanash SM *et al*. 2003. Early changes in gene expression profiles of hepatic GVHD uncovered by oligonucleotide microarrays. *Blood* **102:** (2) 763.

Jiang L, Lindpaintner K, Li HF, Gu NF, Langer H, He L, Fountoulakis M. 2003. Proteomic analysis of the cerebrospinal fluid in patients with schizophrenia. *Amino Acids* **25:** (1) 49.

Kaji T, Hachimura S, Ise W, Kaminogawa S. 2003. Proteome analysis reveals caspase activation in hyporesponsive CD4 T lymphocytes induced *in vivo* by the oral administration of antigen. *J Biol Chem* **278:** (30) 27836.

Kao CY, Chen Y, Zhao YH, Wu R. 2003. ORFeome-based search of airway epithelial cell-specific novel human β-defensin genes. *Am J Respir Cell Mol Biol* **29:** (1) 71.

Kemp TJ, Causton HC, Clerk A. 2003. Changes in gene expression induced by $H_2O_2$ in cardiac myocytes. *Biochem Biophys Res Commun* **307:** (2) 416.

Lee JY, Eom EM, Kim DS, Ha-Lee YM, Lee DH. 2003. Analysis of gene expression profiles of gastric normal and cancer tissues by SAGE. *Genomics* **82:** (1) 78.

Li ZB, Lehar M, Braga N, Westra W, Liu LH, Flint PW. 2003. Study of human laryngeal muscle protein using two-dimensional electrophoresis and mass spectrometry. *Proteomics* **3:** (7) 1325.

Lin ZS, Fillmore GC, Urn TH, Elenitoba-Johnson KJ, Lim MS. 2003. Comparative microarray analysis of gene expression during activation of human peripheral blood T cells and leukemic Jurkat T cells. *Lab Invest* **83:** (6) 765.

Lorenz P, Ruschpler P, Koczan D, Stiehl P, Thiesen HJ. 2003. From transcriptome to proteome: Differentially expressed proteins identified in synovial tissue of patients suffering from rheumatoid arthritis and osteoarthritis by an initial screen with a panel of 791 antibodies. *Proteomics* **3:** (6) 991.

Luo X, Carlson KA, Wojna V, Mayo R, Biskup TM, Stoner J, Anderson J, Gendelman HE, Melendez LM. 2003. Macrophage proteomic fingerprinting predicts HIV-1-associated cognitive impairment. *Neurology* **60:** (12) 1931.

Ma Y, Koza-Taylor PH, DiMattia DA, Hames L, Fu HN, Dragnev KH, Turi T, Beebe JS, Freemantle SJ, Dmitrovsky E. 2003. Microarray analysis uncovers retinoid targets in human bronchial epithelial cells. *Oncogene* **22:** (31) 4924.

Magro G, Perissinotto D, Schiappacassi M, Goletz S, Otto A, Muller EC, Bisceglia M, Brown G, Ellis T, Grasso S *et al*. 2003. Proteomic and postproteomic characterization of keratan sulfate-glycanated isoforms of thyroglobulin and transferrin uniquely elaborated by papillary thyroid carcinomas. *Am J Pathol* **163:** (1) 183.

Manabe T, Yamaguchi N, Mukai J, Hamada O, Tani O. 2003. Detection of protein-protein interactions and a group of immunoglobulin G-associated minor proteins in human plasma by nondenaturing and denaturing two-dimensional gel electrophoresis. *Proteomics* **3:** (6) 832.

Minamino N, Tanaka J, Kuwahara H, Kihara T, Satomi Y, Matsubae M, Takao T. 2003. Determination of endogenous peptides in the porcine brain: Possible construction of peptidome, a fact database for endogenous peptides. *J Chromatogr B* **792:** (1) 33.

Mongodin E, Finan J, Climo MW, Rosato A, Gill S, Archer GL. 2003. Microarray transcription analysis of clinical *Staphylococcus aureus* isolates resistant to vancomycin. *J Bacteriol* **185:** (15) 4638.

Naryzhny SN, Lee H. 2003. Observation of multiple isoforms and specific proteolysis patterns of proliferating cell nuclear antigen in the context of cell cycle compartments and sample preparations. *Proteomics* **3:** (6) 930.

O'Neill KA, Miller FR, Barder TJ, Lubman DM. 2003. Profiling the progression of cancer: Separation of microsomal proteins in MCF10 breast epithelial cell lines using nonporous chromatophoresis. *Proteomics* **3:** (7) 1256.

Oien KA, Vass JK, Downie I, Fullarton G, Keith WN. 2003. Profiling, comparison and validation of gene expression in gastric carcinoma and normal stomach. *Oncogene* **22:** (27) 4287.

Pang ZJ, Xing FQ. 2003. Expression profile of trophoblast invasion-associated genes in the pre-eclamptic placenta. *Br J Biomed Sci* **60:** (2) 97.

Pedersen TX, Leethanakul C, Patel V, Mitola D, Lund LR, Dano K, Johnsen M, Gutkind JS, Bugge TH. 2003. Laser capture microdissection-based *in vivo* genomic profiling of wound keratinocytes identifies similarities and differences to squamous cell carcinoma. *Oncogene* **22:** (25) 3964.

Pellagatti A, Vetrie D, Langford CF, Gama S, Eagleton H, Wainscoat JS, Boultwood J. 2003. Gene expression profiling in polycythemia vera using cDNA microarray technology. *Cancer Res* **63:** (14) 3940.

Pieper R, Gatlin CL, Makusky AJ, Russo PS, Schatz CR, Miller SS, Su Q, McGrath AM, Estock MA, Parmar PP *et al*. 2003. The human serum proteome: Display of nearly 3700 chromatographically separated protein spots on two-dimensional electrophoresis gels and identification of 325 distinct proteins. *Proteomics* **3:** (7) 1345.

Plymoth A, Lofdahl CG, Ekberg-Jansson A, Dahlback M, Lindberg H, Fehniger TE, Marko-Varga G. 2003. Human bronchoalveolar lavage: Biofluid analysis with special emphasis on sample preparation. *Proteomics* **3:** (6) 962.

Poirier F, Joubert-Caron R, Labas V, Caron M. 2003. Proteomic analysis of a lymphoma-derived cell line (DG75) following treatment with a demethylating drug: Modification of membrane-associated proteins. *Proteomics* **3:** (6) 1028.

Ramanathan M. 2003. A stochastic model for optimizing composite predictors based on gene expression profiles. *Pharmaceut Res* **20:** (7) 996.

Sakai J, Ishikawa H, Kojima S, Satoh H, Yamamoto S, Kanaoka M. 2003. Proteomic analysis of rat heart in ischemia and ischemia-reperfusion using fluorescence two-dimensional difference gel electrophoresis. *Proteomics* **3:** (7) 1318.

Sarwal M, Chua MS, Kambham N, Hsieh SC, Satterwhite T, Masek M, Salvatierra O. 2003. Molecular heterogeneity in acute renal allograft rejection identified by DNA microarray profiling. *N Engl J Med* **349:** (2) 125.

Schoneich C. 2003. Proteomics in gerontological research. *Exp Gerontol* **38:** (5) 473.

Segal NH, Pavlidis P, Antonescu CR, Maki RG, Noble WS, DeSantis D, Woodruff JM, Lewis JJ, Brennan MF, Houghton AN *et al*. 2003. Classification and subtype prediction of adult soft tissue sarcoma by functional genomics. *Am J Pathol* **163:** (2) 691.

Seliger B, Menig M, Lichtenfels R, Atkins D, Bukur J, Halder TM, Kersten M, Harder A, Ackermann A, Beck J *et al*. 2003. Identification of markers for the selection of patients undergoing renal cell carcinoma-specific immunotherapy. *Proteomics* **3:** (6) 979.

Shah J. 2003. Economic and regulatory considerations in pharmacogenomics for drug licensing and healthcare. *Nat Biotechnol* **21:** (7) 747.

Shai R, Shi T, Kremen TJ, Horvath S, Liau LM, Cloughesy TF, Mischel PS, Nelson SF. 2003. Gene expression profiling identifies molecular subtypes of gliomas. *Oncogene* **22:** (31) 4918.

Simkhovich BZ, Kloner RA, Poizat C, Majoram P, Kedes LH. 2003. Gene expression profiling: A new approach in the study of myocardial ischemia. *Cardiovasc Pathol* **12:** (4) 180.

Sok JC, Kuriakose MA, Mahajan VB, Pearlman AN, DeLacure MD, Chen FA. 2003. Tissue-specific gene expression of head and neck squamous cell carcinoma *in vivo* by complementary DNA microarray analysis. *Arch Otolaryngol Head Neck Surg* **129:** (7) 760.

Song JH, Kim JM, Kim SH, Kim HJ, Lee JJ, Sung MH, Hwang SY, Kim TS. 2003. Comparison of the gene expression profiles of monocytic *versus* granulocytic lineages of HL-60 leukemia cell differ-

Copyright © 2004 John Wiley & Sons, Ltd. 

entiation by DNA microarray analysis. *Life Sci* **73:** (13) 1705.
Staal FJT, Van der Burg M, Wessels LFA, Barendregt BH, Baert MRM, Van den Burg CMM, Van Huffel C, Langerak AW, Van der Velden VHJ, Reinders MJT, Van Dongen JJM. 2003. DNA microarrays for comparison of gene expression profiles between diagnosis and relapse in precursor-B acute lymphoblastic leukemia: Choice to technique and purification influence the identification of potential diagnostic markers. *Leukemia* **17:** (7) 1324.
Stanley BA, Neverova I, Brown HA, Van Eyk JE. 2003. Optimizing protein solubility for two-dimensional gel electrophoresis analysis of human myocardium. *Proteomics* **3:** (6) 815.
Stulik J, Hernychova L, Porkertova S, Pozler O, Tuckova L, Sanchez D, Bures J. 2003. Identification of new celiac disease autoantigens using proteomic analysis. *Proteomics* **3:** (6) 951.
Swami S, Raghavachari N, Muller UR, Bao YJP, Feldman D. 2003. Vitamin D growth inhibition of breast cancer cells: Gene expression patterns assessed by cDNA microarray. *Breast Cancer Res Treat* **80:** (1) 49.
Taylor CM, Pfeiffer SE. 2003. Enhanced resolution of glycosyl phosphatidylinositol-anchored and transmembrane proteins from the lipid-rich myelin membrane by two-dimensional gel electrophoresis. *Proteomics* **3:** (7) 1303.
Varnum SM, Covington CC, Woodbury RL, Petritis K, Kangas LJ, Abdullah MS, Pounds JG, Smith RD, Zangar RC. 2003. Proteomic characterization of nipple aspirate fluid: Identification of potential biomarkers of breast cancer. *Breast Cancer Res Treat* **80:** (1) 87.
Witzmann FA, Li JY, Strother WN, McBride WJ, Hunter L, Crabb DW, Lumeng L, Li TK. 2003. Innate differences in protein expression in the nucleus accumbens and hippocampus of inbred alcohol-preferring and -nonpreferring rats. *Proteomics* **3:** (7) 1335.
Wu SL, Hancock WS, Goodrich GG, Kunitake ST. 2003. An approach to the proteomic analysis of a breast cancer cell line (SKBR-3). *Proteomics* **3:** (6) 1037.
Zent CS, Zhan FG, Schichman SA, Bumm KHW, Lin P, Chen JB, Shaughnessy JD. 2003. The distinct gene expression profiles of chronic lymphocytic leukemia and multiple myeloma suggest different anti-apoptotic mechanisms but predict only some differences in phenotype. *Leuk Res* **27:** (9) 765.

### 8 EST, cDNA and other clone resources

Bausher M, Shatters R, Chaparro J, Dang P, Hunter W, Niedz R. 2003. An expressed sequence tag (EST) set from *Citrus sinensis* L. Osbeck whole seedlings and the implications of further perennial source investigations. *Plant Sci* **165:** (2) 415.
Carninci P, Waki K, Shiraki T, Konno H, Shibata K, Itoh M, Aizawa K, Arakawa T, Ishii Y, Sasaki D *et al*. 2003. Targeting a complex transcriptome: The construction of the mouse full-length cDNA encyclopedia. *Genome Res* **13:** (6B) 1273.
Hsiang T, Goodwin PH. 2003. Distinguishing plant and fungal sequences in ESTs from infected plant tissues. *J Microbiol Methods* **54:** (3) 339.
Kikuchi S, Satoh K, Nagata T, Kawagashira N, Doi K, Kishimoto N, Yazaki J, Ishikawa M, Yamada H, Ooka H *et al*. 2003. Collection, mapping, and annotation of over 28,000 cDNA clones from japonica rice. *Science* **301:** (5361) 376.
Peng HJ, Chen XG, Wang XZ, Lun ZR. 2003. Analysis of the gene expression profile of *Schistosoma japonicum* cercariae by a strategy based on expressed sequence tags. *Parasitol Res* **90:** (4) 287.
Rossi M, Araujo PG, Paulet F, Garsmeur O, Dias VM, Chen H, Van Sluys MA, D'Hont A. 2003. Genomic distribution and characterization of EST-derived resistance gene analogs (RGAs) in sugarcane. *Mol Genet Genomics* **269:** (3) 406.
Schriml LM, Hill DP, Blake JA, Bono H, Wynshaw-Boris A, Pavan WJ, Ring BZ, Beisel K, Setou M, Okazaki Y. 2003. Human disease genes and their cloned mouse orthologs: Exploration of the FANTOM2 cDNA sequence data set. *Genome Res* **13:** (6B) 1496.
Shimada T, Kita M, Endo T, Fujii H, Ueda T, Moriguchi T, Omura M. 2003. Expressed sequence tags of ovary tissue cDNA library in *Citrus unshiu* Marc. *Plant Sci* **165:** (1) 167.
Torto TA, Li SA, Styer A, Huitema E, Testa A, Gow NAR, Van West P, Kamoun S. 2003. EST mining and functional expression assays identify extracellular effector proteins from the plant pathogen *Phytophthora*. *Genome Res* **13:** (7) 1675.

### 9 Functional genomics

Akilesh S, Shaffer DJ, Roopenian D. 2003. Customized molecular phenotyping by quantitative gene expression and pattern recognition analysis. *Genome Res* **13:** (7) 1719.
Baldarelli RM, Hill DP, Blake JA, Adachi J, Furuno M, Bradt D, Corbani LE, Cousins S, Frazer KS, Qi D *et al*. 2003. Connecting sequence and biology in the laboratory mouse. *Genome Res* **13:** (6B) 1505.
Ebizuka Y, Katsube Y, Tsutsumi T, Kushiro T, Shibuya M. 2003. Functional genomics approach to the study of triterpene biosynthesis. *Pure Appl Chem* **75:** (2-3) 369.
Fathallah-Shaykh HM, He B, Zhao LJ, Engelhard HH, Cerullo L, Lichtor T, Byrne R, Munoz L, Von Roenn K, Rosseau GL *et al*. 2003. Genomic expression discovery predicts pathways and opposing functions behind phenotypes. *J Biol Chem* **278:** (26) 23830.
Game JC, Birrell GW, Brown JA, Shibata T, Baccari C, Chu AM, Williamson MS, Brown JM. 2003. Use of a genome-wide approach to identify new genes that control resistance of *Saccharomyces cerevisiae* to ionizing radiation. *Radiat Res* **160:** (1) 14.
Gardner TS, Di Bernardo D, Lorenz D, Collins JJ*. 2003. Inferring genetic networks and identifying compound mode of action *via* expression profiling. *Science* **301:** (5629) 102.
Nakasono S, Laramee C, Saiki H, McLeod KJ. 2003. Effect of power-frequency magnetic fields on genome-scale gene expression in *Saccharomyces cerevisiae*. *Radiat Res* **160:** (1) 25.
Saunders NFW, Thomas T, Curmi PMG, Mattick JS, Kuczek E, Slade R, Davis J, Franzmann PD, Boone D, Rusterholtz K *et al*. 2003. Mechanisms of thermal adaptation revealed from the genomes of the Antarctic Archaea *Methanogenium frigidum* and *Methanococcoides burtonii*. *Genome Res* **13:** (7) 1580.
Yamamoto YY, Tsuhara Y, Gohda K, Suzuki K, Matsui M. 2003. Gene trapping of the *Arabidopsis* genome with a firefly luciferase reporter. *Plant J* **35:** (2) 273.

### I0 Transcriptomics

Akimoto-Tomiyama C, Sakata K, Yazaki J, Nakamura K, Fujii F, Shimbo K, Yamamoto K, Sasaki T, Kishimoto N, Kikuchi S *et al*. 2003. Rice gene expression in response to *N*-acetylchitooligosaccharide elicitor: Comprehensive analysis by DNA microarray with randomly selected ESTs. *Plant Mol Biol* **52:** (3) 537.
Andersson T, Borang S, Unneberg P, Wirta V, Thelin A, Lundeberg J, Odeberg J. 2003. Shotgun sequencing and microarray analysis of RDA transcripts. *Gene* **310:** 39.
Belland RJ, Zhong GM, Crane DD, Hogan D, Sturdevant D, Sharma J, Beatty WL, Caldwell HD. 2003. Genomic transcriptional profiling of the developmental cycle of *Chlamydia trachomatis*. *Proc Natl Acad Sci U S A* **100:** (14) 8478.
Bono H, Yagi K, Kasukawa T, Nikaido I, Tominaga N, Miki R, Mizuno Y, Tomaru Y, Goto H, Nitanda H *et al*. 2003. Systematic expression profiling of the mouse transcriptome using RIKEN cDNA microarrays. *Genome Res* **13:** (6B) 1318.
Breyne P, Dreesen R, Cannoot B, Rombaut D, Vandepoele K, Rombauts S, Vanderhaeghen R, Inze D, Zabeau M. 2003. Quantitative cDNA-AFLP analysis for genome-wide expression studies. *Mol Genet Genomics* **269:** (2) 173.
Carinci F, Volinia S, Pezzetti F, Francioso F, Tosi L, Piattelli A. 2003. Titanium-cell interaction: Analysis of gene expression profiling. *J Biomed Mater Res* **66B:** (1) 341.
Cowart LA, Okamoto Y, Pinto FR, Gandy JL, Almeida JS, Hannun YA. 2003. Roles of sphingolipid biosynthesis in mediation of specific programs of the heat stress response determined through gene expression profiling. *J Biol Chem* **278:** (32) 30328.
De Chaldee M, Gaillard MC, Bizat N, Buhler JM, Manzoni O, Bockaert J, Hantraye P, Brouillet E, Elalouf JM. 2003. Quantitative assessment of transcriptome differences between brain territories. *Genome Res* **13:** (7) 1646.
Golem S, Culver JN. 2003. Tobacco mosaic virus induced alterations in the gene expression profile of *Arabidopsis thaliana*. *Mol Plant Microbe Interact* **16:** (8) 681.
Gustincich S, Batalov S, Beisel KW, Bono H, Carninci P, Fletcher CF, Grimmond S, Hirokawa N, Jarvis ED, Jegla T *et al*. 2003. Analysis of the mouse transcriptome for genes involved in the function of the ner-

Copyright © 2004 John Wiley & Sons, Ltd. 

vous system. *Genome Res* **13:** (6B) 1395.
Ikehata M, Iwasaka M, Miyakoshi J, Ueno S, Koana T. 2003. Effects of intense magnetic fields on sedimentation pattern and gene expression profile in budding yeast. *J Appl Phys* **93:** (10) 6724.
Jung SH, Lee JY, Lee DH. 2003. Use of SAGE technology to reveal changes in gene expression in *Arabidopsis* leaves undergoing cold stress. *Plant Mol Biol* **52:** (3) 553.
Kepes F. 2003. Periodic epi-organization of the yeast genome revealed by the distribution of promoter sites. *J Mol Biol* **329:** (5) 859.
Lombardi MP, Van den Hoff MJB, Ruijter JM, Luijerink M, Buffing AA, Markman MW, Moorman AFM, Deprez RHL. 2003. Expression analysis of subtractively enriched libraries (EASEL): A widely applicable approach to the identification of differentially expressed genes. *J Biochem Biophys Methods* **57:** (1) 17.
Nikaido L, Saito C, Mizuno Y, Meguro M, Bono H, Kadomura M, Kono T, Morris GA, Lyons PA, Oshimura M *et al*. 2003. Discovery of imprinted transcripts in the mouse transcriptome using large-scale expression profiling. *Genome Res* **13:** (6B) 1402.
Salmon K, Hung SP, Mekjian K, Baldi P, Hatfield GW, Gunsalus RP. 2003. Global gene expression profiling in *Escherichia coli* K12: The effects of oxygen availability and FNR. *J Biol Chem* **278:** (32) 29837.
Sedlak M, Edenberg HJ, Ho NWY. 2003. DNA microarray analysis of the expression of the genes encoding the major enzymes in ethanol production during glucose and xylose co-fermentation by metabolically engineered *Saccharomyces yeast*. *Enzyme Microb Technol* **33:** (1) 19.
Stevenson BJ, Iseli C, Beutler B, Jongeneel CV. 2003. Use of transcriptome data to unravel the fine structure of genes involved in sepsis. *J Infect Dis* **187:** (Suppl 2) S308.
Sudre K, Leroux C, Pietu G, Cassar-Malek I, Petit E, Listrat A, Auffray C, Picard B, Martin P, Hocquette JF. 2003. Transcriptome analysis of two bovine muscles during ontogenesis. *J Biochem* **133:** (6) 745.
Takemoto D, Yoshioka H, Doke N, Kawakita K. 2003. Disease stress-inducible genes of tobacco: Expression profile of elicitor-responsive genes isolated by subtractive hybridization. *Physiol Plant* **118:** (4) 545.
Valenzuela JG, Francischetti IMB, Pham VM, Garfield MK, Ribeiro JMC. 2003. Exploring the salivary gland transcriptome and proteome of the *Anopheles stephensi* mosquito. *Insect Biochem Mol Biol* **33:** (7) 717.
Zavolan M, Kondo S, Schonbach C, Adachi J, Hume DA, Hayashizaki Y, Gaasterland T. 2003. Impact of alternative initiation, splicing, and termination on the diversity of the mRNA transcripts encoded by the mouse transcriptome. *Genome Res* **13:** (6B) 1290.

### II Proteomics

Andon NL, Eckert D, Yates JR, Haynes PA. 2003. High-throughput functional affinity purification of mannose binding proteins from *Oryza sativa*. *Proteomics* **3:** (7) 1270.
Antelmann H, Darmon E, Noone D, Veening JW, Westers H, Bron S, Kuipers OP, Devine KM, Hecker M, Van Dijl JM. 2003. The extracellular proteome of *Bacillus subtilis* under secretion stress conditions. *Mol Microbiol* **49:** (1) 143.
Baglioni P, Bini L, Liberatori S, Pallini V, Marri L. 2003. Proteome analysis of *Escherichia coli* W3110 expressing an heterologous σ factor. *Proteomics* **3:** (6) 1060.
Champion KM, Nishihara JC, Aldor IS, Moreno GT, Andersen D, Stults KL, Vanderlaan M. 2003. Comparison of the *Escherichia coli* proteomes for recombinant human growth hormone producing and nonproducing fermentations. *Proteomics* **3:** (7) 1365.
Emanuelsson O, Elofsson A, Von Heijne G, Cristobal S. 2003. *In silico* prediction of the peroxisomal proteome in fungi, plants and animals. *J Mol Biol* **330:** (2) 443.
Encarnacion S, Guzman Y, Dunn MF, Hernandez M, Vargas MD, Mora J. 2003. Proteome analysis of aerobic and fermentative metabolism in *Rhizobium etli* CE3. *Proteomics* **3:** (6) 1077.
Fortunato D, Giuffrida MG, Cavaletto M, Garoffo LP, Dellavalle G, Napolitano L, Giunta C, Fabris C, Bertino E, Coscia A *et al*. 2003. Structural proteome of human colostral fat globule membrane proteins. *Proteomics* **3:** (6) 897.
Grigoriev A. 2003. On the number of protein-protein interactions in the yeast proteome. *Nucleic Acids Res* **31:** (14) 4157.
Grimmond SM, Miranda KC, Yuan Z, Davis MJ, Hume DA, Yagi K, Tominaga N, Bono H, Hayashizaki Y, Okazaki Y *et al*. 2003. The mouse secretome: Functional classification of the proteins secreted into the extracellular environment. *Genome Res* **13:** (6B) 1350.
Herbert B, Hopwood F, Oxley D, McCarthy J, Laver M, Grinyer J, Goodall A, Williams K, Castagna A, Righetti PG. 2003. β-Elimination: An unexpected artefact in proteome analysis. *Proteomics* **3:** (6) 826.
Huang CM, Foster KW, De Silva T, Zhang JF, Shi ZK, Yusuf N, Van Kampen KR, Elmets CA, Tang DCC. 2003. Comparative proteomic profiling of murine skin. *J Invest Dermatol* **121:** (1) 51.
Jin BF, He K, Wang HX, Wang J, Zhou T, Lan Y, Hu MR, Wei KH, Yang SC, Shen BF *et al*. 2003. Proteomic analysis of ubiquitin-proteasome effects: Insight into the function of eukaryotic initiation factor 5A. *Oncogene* **22:** (31) 4819.
Kanapin A, Batalov S, Davis MJ, Gough J, Grimmond S, Kawaji H, Magrane M, Matsuda H, Schonbach C, Teasdale RD *et al*. 2003. Mouse proteome analysis. *Genome Res* **13:** (6B) 1335.
Kim SY, Kim YS, Bahk YY. 2003. Proteome changes induced by expression of tumor suppressor PTEN. *Mol Cells* **15:** (3) 396.
Kolker E, Purvine S, Galperin MY, Stolyar S, Goodlett DR, Nesvizhskii AI, Keller A, Xie T, Eng JK, Yi E *et al*. 2003. Initial proteome analysis of model microorganism *Haemophilus influenzae* strain Rd KW20. *J Bacteriol* **185:** (15) 4593.
Lafon-Cazal M, Adjali O, Galelotti N, Poncet J, Jouin P, Homburger V, Bockaert J, Marin P. 2003. Proteomic analysis of astrocytic secretion in the mouse: Comparison with the cerebrospinal fluid proteome. *J Biol Chem* **278:** (27) 24438.
Leverrier P, Dimova D, Pichereau V, Auffray Y, Boyaval P, Jan GL. 2003. Susceptibility and adaptive response to bile salts in *Propionibacterium freudenreichii*: Physiological and proteomic analysis. *Appl Environ Microbiol* **69:** (7) 3809.
Marino G, Pucci P, Birolo L, Ruoppolo M. 2003. Exploitation of proteomics strategies in protein structure-function studies. *Pure Appl Chem* **75:** (2-3) 309.
Ndimba BK, Chivasa S, Hamilton JM, Simon WJ, Slabas AR. 2003. Proteomic analysis of changes in the extracellular matrix of *Arabidopsis* cell suspension cultures induced by fungal elicitors. *Proteomics* **3:** (6) 1047.
Pessione E, Giuffrida MG, Prunotto L, Barello C, Mazzoli R, Fortunato D, Conti A, Giunta C. 2003. Membrane proteome of *Acinetobacter radioresistens* S13 during aromatic exposure. *Proteomics* **3:** (6) 1070.
Pferdeort VA, Wood TK, Reardon KF. 2003. Proteomic changes in *Escherichia coli* TG1 after metabolic engineering for enhanced trichloroethene biodegradation. *Proteomics* **3:** (6) 1066.
Plowman JE, Flanagan LM, Paton LN, Fitzgerald AC, Joyce NI, Bryson WG. 2003. The effect of oxidation or alkylation on the separation of wool keratin proteins by two-dimensional gel electrophoresis. *Proteomics* **3:** (6) 942.
Semple CAM. 2003. The comparative proteomics of ubiquitination in mouse. *Genome Res* **13:** (6B) 1389.
Utleg AG, Yi EC, Xie T, Shannon P, White JT, Goodlett DR, Hood L, Lin BY. 2003. Proteomic analysis of human prostasomes. *Prostate* **56:** (2) 150.
Zischka H, Weber G, Weber PJA, Posch A, Braun RJ, Buhringer D, Schneider U, Nissum M, Meitinger T, Ueffing M, Eckerskorn C. 2003. Improved proteome analysis of *Saccharomyces cerevisiae* mitochondria by free-flow electrophoresis. *Proteomics* **3:** (6) 906.

### I3 Metabolomics

Blencke HM, Homuth G, Ludwig H, Mader U, Hecker M, Stulke J. 2003. Transcriptional profiling of gene expression in response to glucose in *Bacillus subtilis*: Regulation of the central metabolic pathways. *Metab Eng* **5:** (2) 133.
Bono H, Nikaido I, Kasukawa T, Hayashizaki Y, Okazaki Y. 2003. Comprehensive analysis of the mouse metabolome based on the transcriptome. *Genome Res* **13:** (6B) 1345.
Goossens A, Hakkinen ST, Laakso I, Seppanen-Laakso T, Biondi S, De Sutter V, Lammertyn F, Nuutila AM, Soderlund H, Zabeau M, Inze D, Oksman-Caldentey KM. 2003. A functional genomics approach toward the understanding of secondary metabolism in plant cells. *Proc Natl Acad Sci U S A* **100:** (14) 8595.
Ismail W, Mohamed ME, Wanner BL, Datsenko KA, Eisenreich W, Rohdich F, Bacher A, Fuchs G. 2003. Functional genomics by NMR spectroscopy: Phenylacetate catabolism in *Escherichia coli*. *Eur J Biochem* **270:** (14) 3047.

Copyright © 2004 John Wiley & Sons, Ltd. 

Plaggenborg R, Overhage J, Steinbuchel A, Priefert H. 2003. Functional analyses of genes involved in the metabolism of ferulic acid in *Pseudomonas putida* KT2440. *Appl Microbiol Biotechnol* **61:** (5-6) 528.
Wong JH, Balmer Y, Cai N, Tanaka CK, Vensel WH, Hurkman WJ, Buchanan BB. 2003. Unraveling thioredoxin-linked metabolic processes of cereal starchy endosperm using proteomics. *FEBS Lett* **547:** (1-3) 151.

### I4 Genomic approaches to development

Arai M, Yokosuka O, Chiba T, Imazeki F, Kato M, Hashida J, Ueda Y, Sugano S, Hashimoto K, Saisho H *et al*. 2003. Gene expression profiling reveals the mechanism and pathophysiology of mouse liver regeneration. *J Biol Chem* **278:** (32) 29813.
Cnudde F, Moretti C, Porceddu A, Pezzotti M, Gerats T. 2003. Transcript profiling on developing *Petunia hybrida* floral organs. *Sex Plant Reprod* **16:** (2) 77.
Helbing CC, Werry K, Crump D, Domanski D, Veldhoen N, Bailey CM. 2003. Expression profiles of novel thyroid hormone-responsive genes and proteins in the tail of *Xenopus laevis* tadpoles undergoing precocious metamorphosis. *Mol Endocrinol* **17:** (7) 1395.
Li TR, White KP. 2003. Tissue-specific gene expression and ecdysone-regulated genomic networks in *Drosophila*. *Dev Cell* **5:** (1) 59.
Madi A, Mikkat S, Ringel B, Thiesen HJ, Glocker MO. 2003. Profiling stage-dependent changes of protein expression in *Caenorhabditis elegans* by mass spectrometric proteome analysis leads to the identification of stage-specific marker proteins. *Electrophoresis* **24:** (11) 1809.
Ok SH, Park HM, Kim JY, Bahn SC, Bae JM, Suh MC, Jeung JU, Kim KN, Shin JS. 2003. Identification of differentially expressed genes during flower development in carnation (*Dianthus caryophyllus*). *Plant Sci* **165:** (2) 291.
Saxena A, Worthey EA, Yan SF, Leland A, Stuart KD, Myler PJ. 2003. Evaluation of differential gene expression in *Leishmania major* Friedlin procyclics and metacyclics using DNA microarray analysis. *Mol Biochem Parasitol* **129:** (1) 103.
Yu ZR, Guo R, Ge YH, Ma J, Guan Jk, Li S, Sun XD, Xue SP, Han DS. 2003. Gene expression profiles in different stages of mouse spermatogenic cells during spermatogenesis. *Biol Reprod* **69:** (1) 37.

### I5 Technological advances

Balazsi G, Kay KA, Barabasi AL, Oltvai ZN ZN. 2003. Spurious spatial periodicity of co-expression in microarray data due to printing design. *Nucleic Acids Res* **31:** (15) 4425
Barczak A, Rodriguez MW, Hanspers K, Koth LL, Tai YC, Bolstad BM, Speed TP, Erle DJ. 2003. Spotted long oligonucleotide arrays for human gene expression analysis. *Genome Res* **13:** (7) 1775.
Beranova-Giorgianni S. 2003. Proteome analysis by two-dimensional gel electrophoresis and mass spectrometry: Strengths and limitations. *Trends Anal Chem* **22:** (5) 273.
Bruschi M, Musante L, Candiano G, Ghiggeri GM, Herbert B, Antonucci F, Righetti PG. 2003. Soft immobilized pH gradient gels in proteome analysis: A follow-up. *Proteomics* **3:** (6) 821.
Burkitt WI, Giannakopulos AE, Sideridou F, Bashir S, Derrick PJ. 2003. Discrimination effects in MALDI-MS of mixtures of peptides: Analysis of the proteome. *Aust J Chem* **56:** (5) 369.
Chen JZ, Balgley BM, DeVoe DL, Lee CS. 2003. Capillary isoelectric focusing-based multidimensional concentration/separation platform for proteome analysis. *Anal Chem* **75:** (13) 3145.
Ding QX, Xiao L, Xiong SX, Jia YF, Que HP, Guo YJ, Liu SJ. 2003. Unmatched masses in peptide mass fingerprints caused by cross-contamination: An updated statistical result. *Proteomics* **3:** (7) 1313.
Du Z, Scott AD, May GD. 2003. Amplification of high-quantity serial analysis of gene expression ditags and improvement of concatemer cloning efficiency. *Biotechniques* **35:** (1) 66.
Frederiksen CM, Aaboe M, Dyrskjot L, Laurberg S, Wolf H, Orntoft TF, Kruhoffer M. 2003. Technical evaluation of cDNA microarrays. *APMIS* **111:** (Suppl 108) 96.
Ghezzi P, Bonetto V. 2003. Redox proteomics: Identification of oxidatively, modified proteins. *Proteomics* **3:** (7) 1145.
Held GA, Grinstein G, Tu Y. 2003. Modeling of DNA microarray data by using physical properties of hybridization. *Proc Natl Acad Sci U S A* **100:** (13) 7575.
Jing D, Agnew J, Patton WF, Hendrickson J, Beechem JM. 2003. A sensitive two-color electrophoretic mobility shift assay for detecting both nucleic acids and protein in gels. *Proteomics* **3:** (7) 1172.
Kawahashi Y, Doi N, Takashima H, Tsuda C, Oishi Y, Oyama R, Yonezawa M, Miyamoto-Sato E, Yanagawa H. 2003. *In vitro* protein microarrays for detecting protein-protein interactions: Application of a new method for fluorescence labeling of proteins. *Proteomics* **3:** (7) 1236.
Knowles MR, Cervino S, Skynner HA, Hunt SP, De Felipe C, Salim K, Meneses-Lorente G, McAllister G, Guest PC. 2003. Multiplex proteomic analysis by two-dimensional differential in-gel electrophoresis. *Proteomics* **3:** (7) 1162.
Koy C, Mikkat S, Raptakis E, Sutton C, Resch M, Tanaka K, Glocker MO. 2003. Matrix-assisted laser desorption/ionization-quadrupole ion trap-time of flight mass spectrometry sequencing resolves structures of unidentified peptides obtained by in-gel tryptic digestion of haptoglobin derivatives from human plasma proteomes. *Proteomics* **3:** (6) 851.
Le Blanc JCY, Hager JW, Ilisiu AMP, Hunter C, Zhong F, Chu I. 2003. Unique scanning capabilities of a new hybrid linear ion trap mass spectrometer (Q TRAP) used for high sensitivity proteomics applications. *Proteomics* **3:** (6) 859.
Lecchi P, Gupte AR, Perez RE, Stockert LV, Abramson FP. 2003. Size-exclusion chromatography in multidimensional separation schemes for proteome analysis. *J Biochem Biophys Methods* **56:** (1-3) 141.
Liska AJ, Shevchenko A. 2003. Combining mass spectrometry with database interrogation strategies in proteomics. *Trends Anal Chem* **22:** (5) 291.
Lolli G, Thaler F, Valsasina B, Roletto F, Knapp S, Uggeri M, Bachi A, Matafora V, Storici P, Stewart A *et al*. 2003. Inhibitor affinity chromatography: Profiling the specific reactivity of the proteome with immobilized molecules. *Proteomics* **3:** (7) 1287.
Lopez MF, Mikulskis A, Golenko E, Herick K, Spibey CA, Taylor I, Bobrow M, Jackson P. 2003. High-content proteomics: Fluorescence multiplexing using an integrated, high-sensitivity, multiwavelength charge-coupled device imaging system. *Proteomics* **3:** (7) 1109.
Luhn S, Berth M, Hecker M, Bernhardt J. 2003. Using standard positions and image fusion to create proteome maps from collections of two-dimensional gel electrophoresis images. *Proteomics* **3:** (7) 1117.
Martin K, Hart C, Liu JX, Leung WY, Patton WF. 2003. Simultaneous trichromatic fluorescence detection of proteins on Western blots using an amine-reactive dye in combination with alkaline phosphatase- and horseradish peroxidase-antibody conjugates. *Proteomics* **3:** (7) 1215.
Martin K, Steinberg TH, Cooley LA, Gee KR, Beechem JM, Patton WF. 2003. Quantitative analysis of protein phosphorylation status and protein kinase activity on microarrays using a novel fluorescent phosphorylation sensor dye. *Proteomics* **3:** (7) 1244.
Masselon C, Pasa-Tolic L, Li LJ, Anderson GA, Harkewicz R, Smith RD. 2003. Identification of tryptic peptides from large databases using multiplexed tandem mass spectrometry: Simulations and experimental results. *Proteomics* **3:** (7) 1279.
Mechin V, Consoli L, Le Guilloux M, Damerval C. 2003. An efficient solubilization buffer for plant proteins focused in immobilized pH gradients. *Proteomics* **3:** (7) 1299.
Miura K. 2003. Imaging technologies for the detection of multiple stains in proteomics. *Proteomics* **3:** (7) 1097.
Petersen A. 2003. Two-dimensional electrophoresis replica blotting: A valuable technique for the immunological and biochemical characterization of single components of complex extracts. *Proteomics* **3:** (7) 1206.
Purvine S, Eppel JT, Yi EC, Goodlett DR. 2003. Shotgun collision-induced dissociation of peptides using a time of flight mass analyzer. *Proteomics* **3:** (6) 847.
Qian JA, Kluger Y, Yu HY, Gerstein M. 2003. Identification and correction of spurious spatial correlations in microarray data. *Biotechniques* **35:** (1) 42.
Raja R, Salunga R, Taylor T, Bennett A, Firouzi A, Mikulowska-Mennis A, Ma XJ, Sgroi D, Erlander M, Kunitake S. 2002. A microgenomics platform for high-throughput gene expression analysis of pure cell populations. *J Clin Ligand Assay* **25:** (3) 253.
Rogers M, Graham J, Tonge PR. 2003. Using statistical image models for objective evaluation of spot detection in two-dimensional gels. *Proteomics* **3:** (6) 879.

Copyright © 2004 John Wiley & Sons, Ltd. 

Rogers M, Graham J, Tonge RP. 2003. Statistical models of shape for the analysis of protein spots in two-dimensional electrophoresis gel images. *Proteomics* **3:** (6) 887.

Rouillard JM, Zuker M, Gulari E. 2003. OligoArray 2.0: Design of oligonucleotide probes for DNA microarrays using a thermodynamic approach. *Nucleic Acids Res* **31:** (12) 3057.

Schulenberg B, Arnold B, Patton WF. 2003. An improved mechanically durable electrophoresis gel matrix that is fully compatible with fluorescence-based protein detection technologies. *Proteomics* **3:** (7) 1196.

Shaw J, Rowlinson R, Nickson J, Stone T, Sweet A, Williams K, Tonge R. 2003. Evaluation of saturation labelling two-dimensional difference gel electrophoresis fluorescent dyes. *Proteomics* **3:** (7) 1181.

Steinberg TH, Agnew BJ, Gee KR, Leung WY, Goodman T, Schulenberg B, Hendrickson J, Beechem JM, Haugland RP, Patton WF. 2003. Global quantitative phosphoprotein analysis using multiplexed proteomics technology. *Proteomics* **3:** (7) 1128.

Toyoda N, Nagai S, Terashima Y, Motomura K, Haino M, Hashimoto S, Takizawa H, Matsushima K. 2003. Analysis of mRNA with microsomal fractionation using a SAGE-based DNA microarray system facilitates identification of the genes encoding secretory proteins. *Genome Res* **13:** (7) 1728.

Wrobel G, Schlingemann J, Hummerich L, Kramer H, Lichter P, Hahn M. 2003. Optimization of high-density cDNA-microarray protocols by 'design of experiments': Online art. no. e67. *Nucleic Acids Res* **31:** (12) e67.

Yan F, Sreekumar A, Laxman E, Chinnaiyan AM, Lubman DM, Barder TJ. 2003. Protein microarrays using liquid phase fractionation of cell lysates. *Proteomics* **3:** (7) 1228.

Yu XL, Wang DS, Wang DX, Ou YJH, Yan Z, Dong Y, Liao W, Zhao XS. 2003. Micro-array detection system for gene expression products based on surface plasmon resonance imaging. *Sensor Actuator B-Chem* **91:** (1-3) 133.

### I6 Bioinformatics

Allison L. 2003. Longest biased interval and longest non-negative sum interval. *Bioinformatics* **19:** (10) 1294.

BarJoseph Z, Demaine ED, Gifford DK, Srebro N, Hamel AM, Jaakkola TS. 2003. K-ary clustering with optimal leaf ordering for gene expression data. *Bioinformatics* **19:** (9) 1070.

Bockhorst J, Craven M, Page D, Shavlik J, Glasner J. 2003. A Bayesian network approach to operon prediction. *Bioinformatics* **19:** (10) 1227.

Bura E, Pfeiffer RM. 2003. Graphical methods for class prediction using dimension reduction techniques on DNA microarray data. *Bioinformatics* **19:** (10) 1252.

Chen LL, Ou HY, Zhang R, Zhang CT. 2003. ZCURVE_CoV: A new system to recognize protein coding genes in coronavirus genomes, and its applications in analyzing SARS-CoV genomes. *Biochem Biophys Res Commun* **307:** (2) 382.

Chu TJ, Glymour C, Scheines R, Spirtes P. 2003. A statistical problem for inference to regulatory structure from associations of gene expression measurements with microarrays. *Bioinformatics* **19:** (9) 1147.

Detours V, Dumont JE, Bersini H, Maenhaut C. 2003. Integration and cross-validation of high-throughput gene expression data: Comparing heterogeneous data sets. *FEBS Lett* **546:** (1) 98.

Dettling M, Buhlmann P. 2003. Boosting for tumor classification with gene expression data. *Bioinformatics* **19:** (9) 1061.

Ding CHQ. 2003. Unsupervised feature selection *via* two-way ordering in gene expression analysis. *Bioinformatics* **19:** (10) 1259.

Dudoit S, Fridlyand J. 2003. Bagging to improve the accuracy of a clustering procedure. *Bioinformatics* **19:** (9) 1090.

Furuno M, Kasukawa T, Saito R, Adachi J, Suzuki H, Baldarelli R, Hayashizaki Y, Okazaki Y. 2003. CDS annotation in full-length cDNA sequence. *Genome Res* **13:** (6B) 1478.

Getz G, Gal H, Kela I, Notterman DA, Domany E. 2003. Coupled two-way clustering analysis of breast cancer and colon cancer gene expression data. *Bioinformatics* **19:** (9) 1079.

Getz G, Domany E. 2003. Coupled two-way clustering server. *Bioinformatics* **19:** (9) 1153.

Hernandez P, Gras R, Frey J, Appel RD. 2003. Popitam: Towards new heuristic strategies to improve protein identification from tandem mass spectrometry data. *Proteomics* **3:** (6) 870.

Horn D, Axel I. 2003. Novel clustering algorithm for microarray expression data in a truncated SVD space. *Bioinformatics* **19:** (9) 1110.

Hudek AK, Cheung J, Boright AP, Scherer SW. 2003. Genescript: DNA sequence annotation pipeline. *Bioinformatics* **19:** (9) 1177.

Hvidsten TR, Laegreid A, Komorowski J. 2003. Learning rule-based models of biological process from gene expression time profiles using gene ontology. *Bioinformatics* **19:** (9) 1116.

Johnson JE, Stromvik MV, Silverstein KAT, Crow JA, Shoop E, Retzel EF. 2003. TableView: Portable genomic data visualization. *Bioinformatics* **19:** (10) 1292.

Jornsten R, Yu B. 2003. Simultaneous gene clustering and subset selection for sample classification *via* MDL. *Bioinformatics* **19:** (9) 1100.

Kasukawa T, Furuno M, Nikaido I, Bono H, Hume DA, Bult C, Hill DP, Baldarelli R, Gough J, Kanapin A *et al*. 2003. Development and evaluation of an automated annotation pipeline and cDNA annotation system. *Genome Res* **13:** (6B) 1542.

Kim PM, Tidor B. 2003. Subsystem identification through dimensionality reduction of large-scale gene expression data. *Genome Res* **13:** (7) 1706.

Lee Y, Lee CK. 2003. Classification of multiple cancer types by tip multicategory support vector machines using gene expression data. *Bioinformatics* **19:** (9) 1132.

Lenhard B, Wahlestedt C, Wasserman WW. 2003. GeneLynx Mouse: Integrated portal to the mouse genome. *Genome Res* **13:** (6B) 1501.

Lord PW, Stevens RD, Brass A, Goble CA. 2003. Investigating semantic similarity measures across the Gene Ontology: The relationship between sequence and annotation. *Bioinformatics* **19:** (10) 1275.

Malde K, Coward E, Jonassen I. 2003. Fast sequence clustering using a suffix array algorithm. *Bioinformatics* **19:** (10) 1221.

Marengo E, Robotti E, Gianotti V, Righetti PG. 2003. A new approach to the statistical treatment of 2D-maps in proteomics using fuzzy logic. *Ann Chim-Rome* **93:** (1-2) 105.

Michaud DJ, Marsh AG, Dhurjati PS. 2003. eXPatGen: generating dynamic expression patterns for the systematic evaluation of analytical methods. *Bioinformatics* **19:** (9) 1140.

Nagashima T, Silva DG, Petrovsky N, Socha LA, Suzuki H, Saito R, Kasukawa T, Kurochkin IV, Konagaya A, Schonbach C. 2003. Inferring higher functional information for RIKEN mouse full-length cDNA clones with FACTS. *Genome Res* **13:** (6B) 1520.

Nakahara H, Nishimura S, Inoue M, Hori G, Amari S. 2003. Gene interaction in DNA microarray data is decomposed by information geometric measure. *Bioinformatics* **19:** (9) 1124.

Ressom H, Wang DL, Natarajan P. 2003. Clustering gene expression data using adaptive double self-organizing map. *Physiol Genomics* **14:** (1) 35.

Shah SP, McVicker GP, Mackworth AK, Rogic S, Ouellette BFF. 2003. GeneComber: Combining outputs of gene prediction programs for improved results. *Bioinformatics* **19:** (10) 1296.

Sun MH, Xiong MM. 2003. A mathematical programming approach for gene selection and tissue classification. *Bioinformatics* **19:** (10) 1243.

Suzuki H, Saito R, Kanamori M, Kai C, Schonbach C, Nagashima T, Hosaka J, Hayashizaki Y. 2003. The mammalian protein-protein interaction database and its viewing system that is linked to the main FANTOM2 viewer. *Genome Res* **13:** (6B) 1534.

Thompson JD, Thierry JC, Poch O. 2003. RASCAL: Rapid scanning and correction of multiple sequence alignments. *Bioinformatics* **19:** (9) 1155.

Troyanskaya OG, Dolinski K, Owen AB, Altman RB, Botstein D. 2003. A Bayesian framework for combining heterogeneous data sources for gene function prediction (in *Saccharomyces cerevisiae*). *Proc Natl Acad Sci U S A* **100:** (14) 8348.

Wit E, McClure J. 2003. Statistical adjustment of signal censoring in gene expression experiments. *Bioinformatics* **19:** (9) 1055.

Xu RH, Li XC. 2003. A comparison of parametric *versus* permutation methods with applications to general and temporal microarray gene expression data. *Bioinformatics* **19:** (10) 1284.

Yang AS, Wang LY. 2003. Local structure prediction with local structure-based sequence profiles. *Bioinformatics* **19:** (10) 1267.

Yang J, Wang JH, Yao ZJ, Jin Q, Shen Y, Chen RS. 2003. GenomeComp: A visualization tool for microbial genome comparison. *J Microbiol Methods* **54:** (3) 423.

Zhang K, Jin L. 2003. HaploBlockFinder: Haplotype block analyses. *Bioinformatics* **19:** (10) 1300.

Zhao YL, Pan W. 2003. Modified nonparametric approaches to detecting differentially expressed genes in replicated microarray experiments. *Bioinformatics* **19:** (9) 1046.

Copyright © 2004 John Wiley & Sons, Ltd. 
